# Investigation of a Model-Based Working Memory Training With and Without Distractor Inhibition and Its Comparative Efficacy: A Randomized Controlled Trial on Healthy Old Adults

**DOI:** 10.3389/fnagi.2021.682474

**Published:** 2021-06-15

**Authors:** Priska Zuber, Emanuel Geiter, Dominique J.-F. de Quervain, Stefano Magon

**Affiliations:** ^1^Division of Cognitive Neuroscience, Faculty of Psychology, University of Basel, Basel, Switzerland; ^2^Transfaculty Research Platform, University of Basel, Basel, Switzerland; ^3^Medical Image Analysis Center (MIAC AG), Basel, Switzerland; ^4^Department of Neurology, University Hospital Basel, University of Basel, Basel, Switzerland; ^5^University Psychiatric Clinics, University of Basel, Basel, Switzerland; ^6^Roche Pharma Research and Early Development, Roche Innovation Center Basel, F. Hoffmann-La Roche Ltd., Basel, Switzerland

**Keywords:** working memory, training, filtering efficiency, distractor inhibition, cognition, aging, cognitive training, practice effects

## Abstract

**Background**: Various working memory (WM) trainings have been tested, but differences in experimental designs, the lack of theoretical background, and the need of identifying task-related processes such as filtering efficiency limit conclusions about their comparative efficacy.

**Objectives**: In this study, we compared the efficacy of a model-based WM training with (MB^+^) and without (MB) distractor inhibition on improving WM capacity to a dual *n-back* and active control condition.

**Methods**: This randomized clinical trial included 123 healthy elderly adults (78 women, 45 men; aged 64.1 ± 8.3 years). All groups underwent 12 40-min training sessions over 3 weeks and four cognitive testing sessions. The first two sessions served as double baseline to account for practice effects. Primary outcome was WM capacity post-training measured by complex span tasks. Near and far transfer was assessed by simple span, n-back, visuospatial and verbal learning, processing speed, and reasoning tasks.

**Results**: Due to preliminary termination (COVID-19), 93 subjects completed the post-training and 60 subjects the follow-up session. On a whole group level, practice effects occurred from prebaseline to baseline in WM capacity (*b* = 4.85, *t*_(103)_ = 4.01, *p* < 0.001, *r* = 0.37). Linear mixed-effects models revealed a difference in WM capacity post-training between MB^+^ and MB (*b* = −9.62, *t*_(82)_ = −2.52, *p* = 0.014, *r* = 0.27) and a trend difference between MB^+^ and dual *n-back* (*b* = −7.59, *t*_(82)_ = −1.87, *p* = 0.065, *r* = 0.20) and control training (*b* = −7.08, *t*_(82)_ = −1.86, *p* = 0.067, *r* = 0.20). Univariate analyses showed an increase between pre- and post-training for WM capacity within MB^+^ (*t*_(22)_ = −3.34, *p* < 0.05) only. There was no difference between groups pre- and post-training regarding near and far transfer. Univariate analyses showed improved visuospatial learning within MB^+^ (*t*_(21)_ = −3.8, *p* < 0.05), improved processing speed (*t*_(23)_ = 2.19, *p*< 0.05) and *n*-back performance (*t*_(23)_ = 2.12, *p* < 0.05) in MB, and improved *n*-back performance (*t*_(25)_ = 3.83, *p* < 0.001) in the dual *n*-back training.

**Interpretation**: A model-based WM training including filtering efficacy may be a promising approach to increase WM capacity and needs further investigation in randomized controlled studies.

## Introduction

Working memory (WM) refers to a cognitive system which temporarily maintains, stores, and manipulates information and acts as an interface between perception, long-term memory, and action (Baddeley, 2003, 2012). This definition emphasizes WM as a top-down mental process, which has been shown to be crucial for higher-order cognitive functions such as problem solving, language comprehension, arithmetic, and decision-making (Diamond, 2013). Thus, impairments of WM due to neurological, psychiatric, and developmental disorder or as a result of aging have a large impact on work performances or daily functioning (Glisky, 2007; Redick, 2019). As a consequence, WM trainings were developed, aiming at increasing WM capacity (Melby-Lervåg et al., 2016; Katz et al., 2017).

Following the first promising results of WM training studies (Klingberg et al., 2005; Jaeggi et al., 2008), the field emerged and resulted in a large body of literature with the common aim of investigating the efficacy of various approaches to improve WM capacity. In experimental studies, the efficacy of WM trainings is typically investigated by the extent of transfer effects, i.e., if possible gains can be generalized to untrained cognitive tasks (Teixeira-Santos et al., 2019). Transfer effects can either occur as near transfer, which describes improvement on untrained tasks similar to the trained tasks (e.g., an untrained WM task), or as far transfer, describing improvement on a task of a different cognitive function (e.g., a fluid intelligence task; Jaeggi et al., 2008; Teixeira-Santos et al., 2019). Although literature agrees on the efficacy of WM trainings in producing near transfer effects, far transfer effects remain a much-debated topic up to date. Indeed, meta-analytic reviews described near transfer effects to verbal and visuospatial WM tasks following WM trainings (Karbach and Verhaeghen, 2014; Melby-Lervåg et al., 2016; Soveri et al., 2017; Teixeira-Santos et al., 2019; Basak et al., 2020), which, moreover, maintained in older healthy adults in the long term (Teixeira-Santos et al., 2019). Although far transfer effects were reported following WM trainings (Soveri et al., 2017), they have been described to be small (Karbach and Verhaeghen, 2014; Teixeira-Santos et al., 2019; Basak et al., 2020), and it has been concluded that there is no convincing evidence for far transfer effects (Melby-Lervåg et al., 2016).

One main challenge in WM training research displays the diversity of training programs, which has been suggested to explain the controversial results of transfer effects (Pergher et al., 2020). So, the type of training has been described as a mediating factor of WM training efficacy (Morrison and Chein, 2011; Teixeira-Santos et al., 2019). Indeed, various WM training approaches have been proposed over the last decades. Whereas strategy-based trainings aim at promoting specific strategies that facilitate the encoding, maintenance, or retrieval of information, multidomain trainings lay their focus on training multiple cognitive functions next to WM with the aim of improving at least one of the trained functions (Morrison and Chein, 2011; Karbach and Verhaeghen, 2014). Process-based WM trainings, however, focus on training the core mechanisms of WM (Morrison and Chein, 2011; Teixeira-Santos et al., 2019) and are usually computerized. Typically, they are either based on simple span tasks targeting the storage component of WM (Klingberg et al., 2005; Könen et al., 2016) or complex span tasks that target the updating, binding, and processing function of WM (Redick and Lindsey, 2013). While span trainings typically include multiple task, the “dual *n*-back training” (Jaeggi et al., 2008) is a widely used process-based WM training assuming that visual and auditory WM are trained concurrently in one single task using auditory and visuospatial stimuli (Morrison and Chein, 2011). However, it has been concluded that using different training tasks on multiple components of WM is more effective in producing near and far transfer gains compared with single-task trainings (Basak et al., 2020). The vast number of training paradigms may explain varying transfer effects, but hamper the possibility of comparing studies (Pergher et al., 2020). For this reason, recent WM training studies attempt to overcome this issue by investigating different training paradigms simultaneously to study their comparative efficacy. A direct comparison of a spatial *n-back* and a verbal complex span training showed near transfer from the *n-back* trainings to a new form of *n-back* task only, whereas no transfer was found for complex span trainings (Minear et al., 2016; Holmes et al., 2019). Furthermore, it has been described that *n-back* and complex span trainings do differ not only in their effectiveness on transfer but also in their underlying neural mechanisms (Blacker et al., 2017).

Additional factors have been identified to explain the controversial evidence of transfer effects. For instance, not all studies included active control groups undergoing a sham intervention in their design even though this has been suggested to be crucial in order to lead to evidence that the WM training is causal for improvements in untrained tasks (Melby-Lervåg et al., 2016). Furthermore, the repeated administration of WM capacity tests to assess training gains has been shown to lead to practice effects (also referred to as retest or learning effects), which could overestimate the actual net training gains of WM trainings (Scharfen et al., 2018). Although high practice effects have been described from the first to the second test administration (Scharfen et al., 2018), their control is mostly neglected in study designs. Moreover, the fact that most WM training programs were not based on theoretical models may have hampered optimal treatment effects and the understanding of the underlying mechanisms (Bastian et al., 2013). Indeed, a WM training based on Baddeley’s WM model focusing on storage, selective attention, and central executive processes was tested on its efficacy in a clinical trial in healthy old adults (Weicker et al., 2018). Each subprocess of WM was trained by a task to remember cards (storage module), focus on specific aspects of cards (selective attention module), and sort cards (manipulation module). The authors reported an increased WM performance on untrained tasks and an increase in everyday life activities; however, no far transfer was described, which was explained through the limited diverseness of the used training tasks (Weicker et al., 2018). Despite no far transfer was found, only a model-based structure of the training allows to draw conclusions on underlying processes that were trained and to identify task-related processes that may induce far transfer effects.

At last, task-related processes have been described to improve WM performance following training (Minear et al., 2016). Indeed, inhibitory abilities such as filtering efficiency—the ability to exclude irrelevant information from assessing WM (Li et al., 2017)—have been shown to render WM more efficiently than WM training alone (Schmicker et al., 2016). It has been described that older adults have difficulties to suppress task-irrelevant information during visual WM encoding, and thus, WM trainings are needed that train the exclusion of irrelevant information during WM encoding (Gazzaley et al., 2008; Jost et al., 2011). Indeed, a comparative study showed the same extent of improvement on WM tasks following filtering training on distractor inhibition compared with a WM training only (Schmicker et al., 2016). Nevertheless, only one study addressed far transfer following filtering efficiency training and did not find effects (Li et al., 2017). Additionally, although training of filtering efficiency has been associated with increased visual WM (Li et al., 2017), its effect on verbal WM has not been investigated yet. Thus, in order to understand if suppression of irrelevant information could display a task-related process for transfer effects following WM training, filtering efficiency should be embedded in verbal and visuospatial WM tasks. So far, no WM training study implemented filtering efficiency in WM tasks.

In summary, various approaches have been tested, but no clear conclusion about their comparative efficacy can be drawn. The main cause of this uncertainty relates to the lack of theoretical background, differences in terms of experimental designs, and the need of identifying task-related processes. In this parallel group randomized clinical trial, we aimed at testing the efficacy of WM training based on Baddeley’s multicomponent model (Baddeley et al., 1974; Baddeley, 2003; Baddeley et al., 2011), which additionally trains filtering efficiency by: (1) embedding it in the WM training and (2) targeting both verbal and visuospatial modalities. Whereas most WM trainings include tasks for training the phonological loop, visuospatial sketchpad, and central executive, our training additionally includes a task for the episodic buffer resulting in a model-based WM training (MB). We implemented novel task levels that target filtering efficiency in the context of WM and added them to the MB training (MB^+^). Both trainings (MB and MB^+^) will be tested for their efficacy in improving WM performance by comparing them to a dual *n*-back training (Jaeggi et al., 2008) and an active control group. In order to minimize the learning effects related to the repetition of the assessment, a double-baseline design is implemented as suggested previously (McCaffrey and Westervelt, 1995; Duff et al., 2001). We hypothesize that the MB^+^ training shows superiority in improving WM performance and inducing transfer effect compared with a dual *n-back* training and an active control group.

## Materials and Methods

### Participants

Based on our power calculations, we targeted to include a total of 120 subjects as the final sample with complete study termination, yielding 30 subjects in each of the four intervention groups to reach a moderate effect size (power = 0.8 and alpha = 0.05, two-sided). Subjects were recruited through online advertisement, advertisement in public transportation, and courses for seniors at the University of Basel. Inclusion criteria were the presence of an informed consent as documented by signature and age 50 years old or older. Participants were excluded if they had: (1) a medical history of psychiatric or neurological disorder assessed with a health status questionnaire and the Montgomery Åsperg Depression Rating Scale (MADRS; Montgomery and Asberg, 1979), (2) a history of substance abuse, (3) a benzodiazepine intake on a daily basis, (4) a color vision deficiency defined by less than 13 correct answers at the Ishihara test (Ishihara, 1987), (5) disability of the upper limbs that limits the use of tablet devices, or (6) less than 26 points on the Montreal Cognitive Assessment (MoCA; Nasreddine et al., 2005). Written informed consent was obtained from each participant after a detailed explanation of the study procedures. The study was approved by the local ethics committee (Ethikkommission Nordwest und Zentralschweiz) and was conducted in accordance with the Declaration of Helsinki. Participants were reimbursed with CHF 100 for their participation.

#### Experimental Design and Procedures

**Figure 1:**
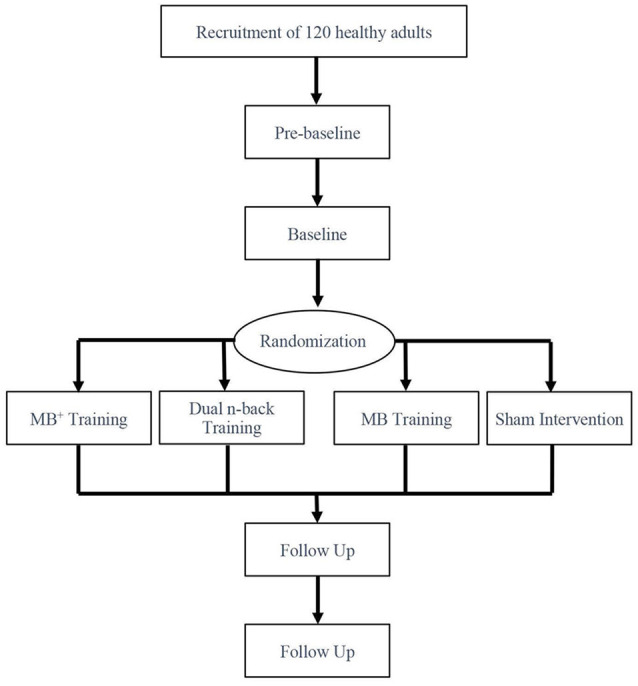
Overview of the study design.

A longitudinal, parallel group, randomized controlled trial design was employed. Although the study was designed as double blind and participants had no information about the performed training, it is not possible to fully blind the participants toward detecting which training group they were allocated to. For this reason, the study is referred to as single blind. After initial recruitment over the phone, participants were invited to our institute for a 30-min screening session where the eligibility criteria were verified for each participant and written informed consent was obtained. After inclusion in the study, all participants underwent four cognitive assessment sessions. The first two sessions (prebaseline and baseline) took place within 3 weeks. At the second session (baseline), participants were randomly allocated to one of the four experimental groups using a minimization approach stratifying the sample regarding age and education. At baseline, participants received the tablet device with the corresponding training as well as a detailed explanation of it. To ensure the single blindness of the study, the study personnel were divided into training explanators, which explained the tablet and the training and provided a short example of each task to the subjects, and cognitive testers. Training explanators were not involved in any procedures addressing the cognitive testing. After the second session, participants were asked to train 3 weeks on the tablet devices at home. After 3 weeks and the completion of the training program, participants underwent a third assessment session to capture possible training effects. An additional assessment session was performed at 12 weeks after the completion of training to investigate long-term effects. Each assessment session took place in a single subject setting and had a duration of approximately 2 h (Figure 1).

#### Trainings

The participants performed the trainings at home using touch-screen tablet devices provided by the research group. All trainings were implemented using Java in Android Studio v1.5.1^1^ and downloaded on Lenovo TAB A10 with Android 4.4 as operating system. All tasks were based on an adaptive design where difficulties of the tasks were modulated according to the participants’ performance. In order to guarantee a comparability of the trainings, the four training regimens were created by using the same visual design, length of the training session, and number of sessions. All participants were instructed to train for 3 weeks, four sessions a week, 45 min each. In order to assess adherence to this training regimen, the date and time of each training were logged and participants were additionally asked to note the date and time of completed training in a diary. Participants were allowed to choose the 4 days of training in 1 week; however, the training was programmed in a way that only one training session was possible each day. Participants were included in the final analysis upon completion of a minimum of 80% of the training (nine training sessions).

#### MB and MB^+^ Training

Our in-house-developed model-based WM trainings aimed at training participants on visuospatial and verbal WM as well as the central executive and episodic buffer function of WM on the theoretical ground of the multicomponent model (Baddeley et al., 1974).

Based on literature review, for each component—phonological loop, visuospatial sketchpad, episodic buffer, and central executive—the task with the highest reliability and validity was chosen as the basis for the MB and MB^+^. For both trainings, the assessment tasks were then transformed into a training task by creating adaptive levels of difficulty based on the number of items and speed. For the MB^+^, additional levels were created based on distractor inhibition corresponding to filtering efficiency for the visuospatial sketchpad and phonological loop tasks (Baddeley, 2012; Li et al., 2017). Table 1 provides an overview over tasks and levels of the MB and MB^+^ trainings. For the phonological loop, a simple letter span test was used as the basis of the training task. For the MB training, increasing item length was used as the level of difficulty. In order to incorporate distractor inhibition for the MB^+^ training: (1) the presence of irrelevant background sounds on the ground of the “irrelevant noise effect” and (2) the presentation of dissimilar or similar digits of items following the “similarity effect” (Baddeley, 2003) were added to the increased sequence length as additional difficulty levels. For the visuospatial sketchpad, a visual pattern span test (Sala et al., 1999) was the basis for the visuospatial subcomponent. In this task, participants were asked to recall the pattern of colored squares in a grid by filling in the right positions in an empty grid, whereas difficulty increased with the size of the grid. For the MB^+^ training, a level of difficulty was added by including an irrelevant visually loaded picture during the retention phase and the recall of the grid squares. A Corsi block-tapping test (Corsi, 1972; Baddeley, 2003) formed the basis for the spatial subcomponent. In this task, participants had to recall flashing objects in the same order they appeared in the arrangement by tapping on the objects. The task improved in difficulty by increasing the item sequence. For the MB^+^ training, a haptic irrelevant movement task was added between the presentation and recall of the object sequence task to the increasing sequence length of the presented items. The central executive component was targeted with a dual task in order to address the ability to focus, divide, and switch attention (Logie et al., 2000; Mohr and Linden, 2005). For the dual task, participants were asked to complete a visual recall task and simultaneously a phonological task of recognizing high and low tones. Difficulty increased by increasing the speed and number of items. For the episodic buffer, we implemented a unimodal binding task where a spatial order of letters embedded in a frame is presented on a stimulus slide. Participants were asked to remember the exact position and letter and recall both in probe slides. Increased difficulty was achieved by adding letter/position combinations to the stimulus slide. The central executive and episodic buffer task were the same for both the MB and MB^+^ trainings.

**Table 1:** Representation of training tasks according to each working memory (WM) component of the multicomponent model (Baddeley, 2012) and levels of difficulty for the MB and MB^+^ WM training.

	Phonological loop (digit span)	Visuospatial sketchpad (pattern span and corsi)		Central executive (dual task)	Episodic buffer (binding task)
MB	Increasing sequence length	Increasing matrx size	Increasing sequence length	Increasing number of items	Increasing number of probes
	Adaptive	Adaptive	Adaptive	Adaptive	Adaptive
MB^+^	MB + irrelevant noise effect + similarity effect	MB + irrelevant picture effect	MB + irrelevant movement task	MB	MB

*Notes. MB, model-based WM training; MB^+^, model-based WM training with distractor inhibition*.

##### Dual *N*-Back Training

The basis for the dual *n*-back training was the widely used “dual *n-back* training paradigm” (Jaeggi et al., 2008). A complex dual *n-back* task including a visual and an auditory WM task was implemented according to the original publication, fit to tablet devices and designed to assure comparability with the other trainings. We used our implemented graphic items instead of squares for the visuospatial task and letters in German language. Except for these adaptions, all parameters on item presentation and retention phases were kept as implemented in the original version.

##### Control Training

As a last comparator group, we included an active control intervention, since active control groups have been suggested to be more reliable in order to prove the specificity of WM training effects (Weicker et al., 2016) and have the function to be able to control for intervention and Hawthorne effects (Bastian et al., 2014). The control intervention in our study was as well developed in-house and consisted of three training tasks addressing manual dexterity, visual–motor coordination, and fine motor control. For the manual dexterity task, subjects had to execute a series of finger movements following a visual cue and were asked to touch circles on the tablet screen that will change color with the corresponding finger. With increasing level, the speed of the presented visual cue and the number of cues increased. For the visual–motor coordination task, the participants are asked to follow the lines of presented letters which they heard also through headphones. With increasing difficulty, the letters were displayed incomplete and the subjects had to complete the presented letter which they heard by drawing them on the tablet. For the fine motor control task, the participants were asked to erase presented moving objects on the screen by executing a swishing movement using the index finger in a dedicated strip at the bottom of the screen. With increasing difficulty, the number of objects moved from the top of the screen to the bottom increased as well as the speed of the objects moving down.

##### Expectation Toward Improvement

The comparability of the four trainings and the expectations toward the improvement in the main outcomes of the different trainings were tested in a separate group of subjects not included in the trial. Volunteers aged around 50 years or older were asked to test the training for a few days and give feedback about their expectation for improving in WM tasks following the training. After a detailed explanation of the training and the main outcome measures, we asked a total of 20 people (five for each training group) in the similar age range of the target sample (*M* = 60.58, SD = 10.35) on how much they would expect to improve on the main outcome after perceiving the training. They had to rate their improvement on a 10-point Likert scale from 0 = “I will not improve in this task by this training at all” to 10 = “I will improve in this task through this training very much”. A Kruskal–Wallis test was applied to investigate if the groups differ in their expectancy ratings. The results yielded no significant differences between the groups, indicating that participants had similar expectations toward the efficacy of the training and would therefore not detect the control training as such.

#### Cognitive Assessment

Cognitive assessment was performed at all four sessions in order to assess possible improvements on WM as well as near and far transfer. To cover a spectrum of transfer tasks, the assessment of cognitive functions included standardized tests (both pencil and article as well as computerized tests) addressing WM, verbal and visual learning, processing speed, and fluid intelligence. Parallel test forms were applied for tests investigating memory recall to further account for learning effects in the applied items. The assessments were performed at the Division of Cognitive Neuroscience by trained psychologists.

#### Working Memory

To investigate the near transfer of the trainings to WM, tasks were included that assess the storage, rehearsal, and processing functions of WM. In old age, it has been shown that age-related effects in complex WM span tasks are higher than in single span tasks, since complex WM span tasks require the coordination of concurrent storage and processing, which is absent in single span tasks. Indeed, larger decreases in performance have been shown for complex WM span tasks than for single span tasks (Bopp and Verhaeghen, 2005). In addition, it has been stated that a more “pure” measure of WM capacity can be derived from using three complex span tasks than from only using one measure to assess WM (Conway et al., 2005). For these reasons, WM transfer as a main outcome was assessed using the shortened versions of the rotation span task, symmetry span task, and operation span task (Foster et al., 2014). It has further been shown that the complex WM span tasks measure a domain-general capacity of WM and highly correlate with each other despite the altering content of the single task (Foster et al., 2014). For this reason, already in previous studies, complex WM tasks were translated into a composite score (Borella and Carretti, 2008; Chiaravalloti et al., 2015). In our study, we similarly created a composite score out of the operation, rotation, and symmetry span tasks by building a sum of the partial score of each task, which then was used as the main outcome in the statistical model.

#### Near Transfer

In order to exploratory investigate if possible training gains are limited to the underlying tasks of the model-based trainings, we included the Corsi block-tapping test which assesses visuospatial WM (Corsi, 1972) as well as a visuospatial *n*-back task (Bürki et al., 2014) and the forward and backward digit span (WAIS-IV; Wechsler, 2008) task which assesses auditory WM. Near transfer to WM-related cognitive functions such as verbal learning was assessed using the Rey Auditory Verbal Learning Test (RAVLT; Schmidt, 1996), to recall of nonverbal information by the Rey Osterrieth Complex Figure Test (ROCFT; Rey, 1941; Osterrieth, 1944), and to executive functions by the Trail Making Test forms A and B (Reitan, 1958).

#### Far Transfer

Reasoning abilities were assessed using Raven’s Standard Progressive Matrices (SPM; Raven and Court, 1996). In order to reduce the administration time, a nine-item short form of the SPM has been created and extensively tested on its psychological property in healthy controls and patients with schizophrenia. Results revealed a correlation of *r* = 0.9836 (form A) and *r* = 0.9782 (form B) with the original 60-item form of the SPM which allow the authors to conclude that the properties of the short forms are comparable with the original form of the SPM (Bilker et al., 2012). As a last secondary outcome measures, the Depression, Anxiety and Stress Scale (DASS-42; Lovibond and Lovibond, 1995) was used to assess depression, anxiety, and stress symptoms.

#### Statistical Analysis

Demographic factors were compared among groups using ANOVAs and chi-square test. Baseline differences were analyzed using an ANOVA model with baseline performance as outcome and group as the between-group factor. In order to investigate possible repetition effects between the prebaseline and baseline sessions, a linear mixed-effects model was carried out on the whole group level with session as the within-subject factor on test performance on all tasks.

In order to analyze the training gains in all outcome tasks, pre- and post-training sessions of the outcome measures were analyzed using a linear mixed-effects model with the interaction term session × training group as fixed effect and study participant as random effect. An advantage of applying the linear mixed-effects models in the analyses of longitudinal data is the ability to account for missing data points (Krueger and Tian, 2004). For this reason, subjects were also included in the analyses if they did not complete all sessions due to the early termination of the study. In order to investigate the superiority to other training approaches, the MB^+^ training was set as baseline comparator using an *a priori* contrast. F statistics were gained by running an ANOVA over the linear model using type II sums of squares. The same statistical analyses were applied for the long-term gain analyses between the post-training and follow-up session at 3 months. In order to investigate differences in means pre- and post-training and post-training to follow-up, univariate analyses within the groups were done on all complete cases using paired *t*-tests. For each statistical analysis, a *p*-value < 0.05 was considered as significant. All data were analyzed in R Studio, Version 1.2.1335 (R Core Team, 2013).

### Results

**Figure 2:**
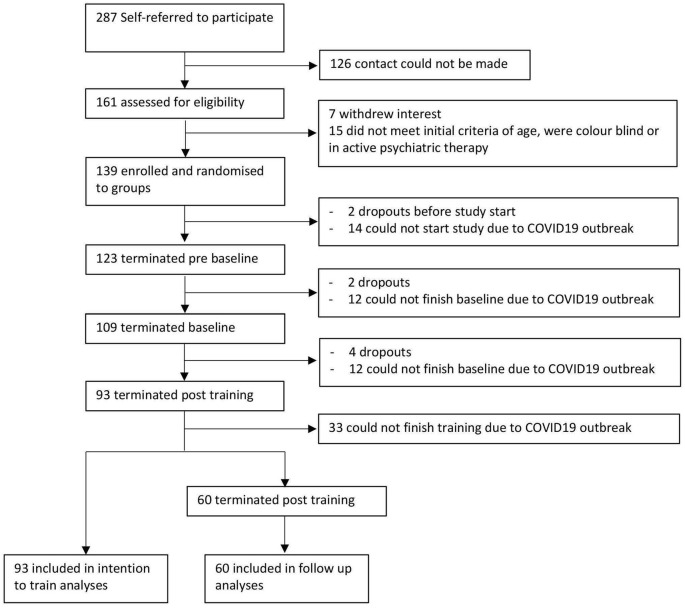
Flowchart of participant recruitment and study inclusion.

Due to the coronavirus disease 2019 (COVID-19) pandemic and, subsequently, the unplanned early determination of the study, the aimed sample size could not be reached. Between July 02, 2019, and the early termination on March 13, 2020, 161 participants from the German-speaking part of Switzerland were screened for participation, of which 38 in total either did not meet the eligibility criteria, withdrew interest before the study start, or could not start due to the COVID-19 outbreak. One hundred and twenty-three healthy subjects aged 50–81 (78 women and 45 men; aged 64.1 ± 8.3 years) were included in the study and completed the prebaseline session. One hundred and nine (68 women and 41 men; aged 64.5 ± 8.2 years) completed the prebaseline and the baseline sessions. 93 subjects (59 women and 34 men; mean age: 64.3 ± 7.8 years) completed the prebaseline, baseline, and post-training sessions as well as the training period. Out of this sample, 60 subjects completed the whole study participation (42 women and 18 men; mean age: 64.2 ± 7.2 years). Figure 2 shows the trial profile and included sample. Demographics of the included sample at prebaseline are listed in Table 2. All 123 subjects were randomized to one of the four training groups after the prebaseline yielding an *n* = 29 in the MB^+^, *n* = 32 in the MB, *n* = 33 in the dual *n-back* training group, and *n* = 29 in the control intervention group. All groups did not differ (*p* < 0.05) in age, years of education, MoCA, MADRS, and Ishihara score at prebaseline.

**Table 2:** Demographic characteristics of the included sample at prebaseline.

	*N*	Sex (f, m)	Education (*M* ± SD)	Age (*M* ± SD)	MoCA (*M* ± SD)	MADRS (*M* ± SD)	Ishihara(*M* ± SD)
MB^+^ training	29	17,12	15.7 ± 3.5	64.3 ± 8.4	28.1 ± 1.3	0.7 ± 1.3	20.4 ± 3.8
MB training	32	21,11	16.4 ± 4.0	64.5 ± 8.8	28.4 ± 1.1	0.6 ± 1.0	21.7 ± 0.7
Control training	29	21,8	15.3 ± 3.4	63.5 ± 8.1	28.3 ± 2.0	0.7 ± 1.1	21.9 ± 1.8
Dual *n*-back training	33	19,14	15.1 ± 3.2	64.0 ± 8.2	28.2 ± 1.2	0.9 ± 1.6	21.5 ± 1.1
Total	123	78,45	15.6 ± 3.5	64.1 ± 8.2	28.3 ± 1.4	0.7 ± 1.2	21.4 ± 2.2

### Training Data

Ninety-three participants received a tablet for training and were instructed to train four times a week for 3 weeks. Two participants were excluded for the analyses of pre- and post-training performance since one participant completed three and another one 24 training sessions. In average, participants completed 11.82 ± 0.81 training sessions. Seventy-five out of 92 subjects (81.5%) completed 12 training sessions; however, 90 subjects (97.8%) of the included 92 participants completed (for inclusion) the required 80% of the training (nine or more training sessions).

### Analyses of Baseline Performance at the Whole Group Level

Results indicated no difference in baseline performance for the complex span score between all groups in neither the prebaseline (*F*_(3,115)_ = 1.60, n.s.) nor the baseline session (*F*_(3,102)_ = 1.04, n.s.) for the complex span composite score.

Regarding the near transfer tasks, no difference between all groups in baseline performance neither at the prebaseline nor baseline was present. For the other outcomes of interest, only the digit span task showed a significant group difference between groups at the prebaseline (*F*_(3,119)_ = 3.52, *p* < 0.05); however, the group difference was not present at the baseline session. For the Corsi block-tapping test and the *n*-back test, groups did not differ in their performance at prebaseline or baseline.

### Analyses of Practice Effects Between Double Baseline at the Whole Group Level

A linear mixed-effects model comparing the prebaseline to the baseline performance in the complex span composite score revealed a significant effect for session ($\chi_{\left(1\right)}^{2}$ = 16.20, *p* < 0.001), indicating a repetition effect. On the whole group level, there was a significant increase in performance on the complex span composite score at baseline compared with prebaseline (*b* = 4.85, *t*_(103)_ = 4.01, *p* < 0.001, *r* = 0.37), despite no training took place between these sessions.

Regarding the transfer tasks, there were significant effects for session in the 30-min recall of the ROCFT ($\chi_{\left(1\right)}^{2}$ = 19.4, *p* < 0.001) with a significant increase in performance at baseline compared with prebaseline (*b* = 2.07, *t*_(108)_ = 4.38, *p* < 0.001, *r* = 0.39), the TMT form B ($\chi_{\left(1\right)}^{2}$ = 18.37, *p* < 0.001) with a significant decrease in reaction time at baseline compared with prebaseline (*b* = −9.61, *t*(108) = −4.27, *p* < 0.001, *r* = 0.38), the *n-back* task ($\chi_{\left(1\right)}^{2}$ = 10.51, *p* < 0.01) with a significant decrease in wrong answers at baseline compared with prebaseline (*b* = −2.92, *t*_(106)_ = −3.23, *p* < 0.001, *r* = 0.30), the DASS anxiety subscale ($\chi_{\left(1\right)}^{2}$ = 4.80, *p* < 0.05) with a significant decrease in score at baseline compared with prebaseline (*b* = −0.37, *t*_(106)_ = −2.18, *p* < 0.05, *r* = 0.21), the DASS depression subscale ($\chi_{\left(1\right)}^{2}$ = 5.59, *p* < 0.05) with a significant decrease in score at baseline compared with prebaseline (*b* = −0.55, *t*_(106)_ = −2.35, *p* < 0.05, *r* = 0.22), and the DASS stress subscale ($\chi_{\left(1\right)}^{2}$ = 4.52, *p* < 0.05) with a significant decrease in score at baseline compared with prebaseline (*b* = −0.79, *t*_(106)_ = −2.12, *p* < 0.05, *r* = 0.20). No changes between the two assessment sessions were found for the RAVLT, the SPM, the Corsi test, and the digit span test. All means and standard deviations of the prebaseline and baseline session are displayed in Table 3.

**Table 3:** Means (*M*), standard deviation (SD), and test statistics comparing prebaseline and baseline session on all outcome measures on the whole group level.

	Prebaseline (*M* ± SD)	Baseline (*M* ± SD)	Prebaseline vs. baseline (*p*)	Effect size (*r*)
Complex span composite score	50.65 ± 18.45	54.77 ± 19.38	<0.001	0.37
Near transfer
ROCFT 30 min recall	20.83 ± 6.04	22.81 ± 5.56	<0.001	0.39
RAVLT 30 min recall	10.98 ± 3.13	11.17 ± 2.84	n.s.	–
TMT B (s)	84.45 ± 30.22	75.69 ± 27.75	<0.001	0.38
Far transfer
SPM	6.13 ± 2.13	6.06 ± 1.86	n.s.	–
DASS depression	2.22 ± 3.28	1.64 ± 2.84	<0.05	0.22
DASS anxiety	1.44 ± 1.90	1.06 ± 1.76	<0.05	0.21
DASS stress	5.45 ± 4.69	4.67 ± 5.29	<0.05	0.20
Other outcomes of interest
Digit span	14.82 ± 3.46	15.07 ± 3.25	n.s.	–
Corsi block	14.24 ± 3.36	14.59 ± 3.38	n.s.	–
*n*-back (wrong answers)	18.37 ± 9.02	15.88 ± 10.77	<0.01	0.30

*Notes. ROCFT, the Rey Osterrieth Complex Figure Test; RAVLT, Rey Auditory Verbal Learning Test; TMT B, Trail Making Test form B; SPM, Standard Progressive Matrices; DASS, Depression Anxiety and Stress Scale; n.s., not significant*.

### Training Effects on the Complex Span Composite Score

**Figure 3:**
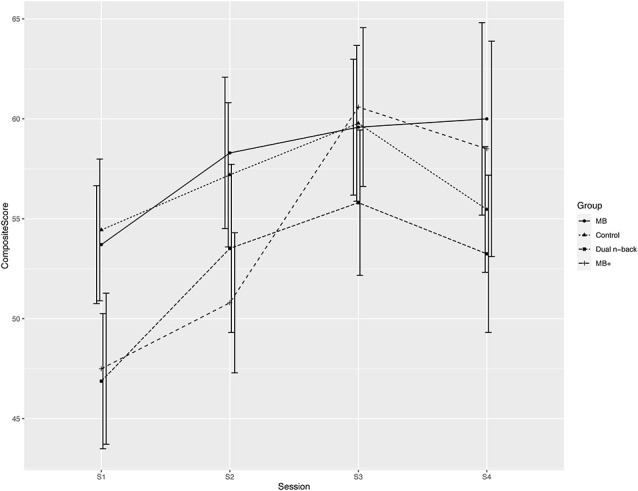
Mean and standard error of the complex span composite score for each of the model-based (MB), control, dual n-back, and model-based plus (MB^+^) training groups over the four sessions.

The linear mixed-effects model revealed a significant main effect for session ($\chi_{\left(1\right)}^{2}$ = 10.56, *p* = 0.001) and a tendency for significance in the interaction group × session ($\chi_{\left(3\right)}^{2}$ = 7.29, *p* = 0.063) for the composite score between baseline and post-training. Setting the MB^+^ group as an *a priori* contrast showed that there was a significant difference post-training between the MB^+^ and the MB training (*b* = −9.62, *t*_(82)_ = −2.52, *p* = 0.014, *r* = 0.27) and a tendency for significance in the comparison between the MB^+^ and the dual *n-back* training (*b* = −7.59, *t*_(82)_ = −1.87, *p* = 0.065, *r* = 0.20) and the control intervention (*b* = −7.08, *t*_(82)_ = −1.86, *p* = 0.067, *r* = 0.20). From post-training to the follow-up at 3 months, the linear mixed-effects model showed no significant effects for group ($\chi_{\left(3\right)}^{2}$ = 1.56, *p* = n.s.), session ($\chi_{\left(1\right)}^{2}$ = 0.40, *p* = n.s.), or the interaction between group and session ($\chi_{\left(3\right)}^{2}$ = 2.99, *p* = n.s.) for the complex span composite score. Figure 3 illustrates the mean of the complex span composite score at all four sessions for all groups.

**Figure 4:**
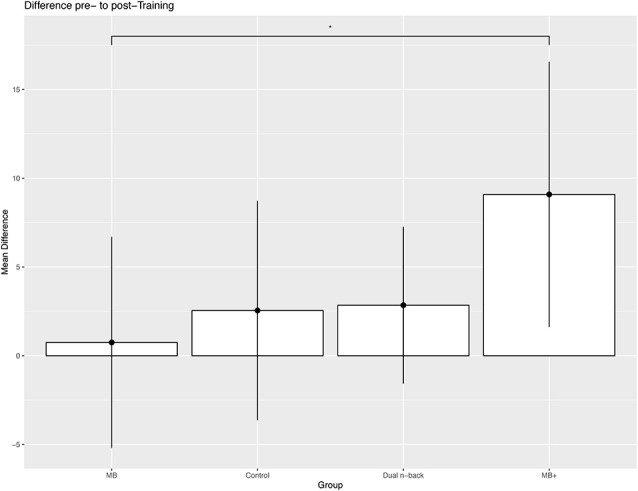
Representation of the mean difference of the complex span composite score between pre- and post-training in all training groups.

Results of univariate analyses showed a significant within-group difference between the pre-and post-training session in the MB^+^ training group (*t*_(22)_ = −3.34, *p* < 0.05). All other trainings and the sham interventions showed no differences in pre- and post-training on the complex span composite score in univariate analyses (Figure 4). Results show no significant group effect for the difference of the complex span composite score between post-training and follow-up after 3 months. Also, the univariate analyses showed no significant differences in the composite score between the post-training and follow-up in the MB^+^, MB, and dual *n*-back training. However, the univariate analyses of the control training showed a significant decrease in performance on the composite score from the post-training session to the follow-up (*t*_(13)_ = 2.56, *p* < 0.05). Mean, standard deviation, and effect sizes for the univariate within-group analyses are reported in Table 4.

**Table 4:** Means, standard deviations, and univariate comparisons of the neuropsychological assessment at each session for each group.

	Pre-training	Post-training	Follow-up	Pre- vs. post-training		Post-training vs. follow-up	
				*p*	*d*	*p*	*d*
**MB^+^**
Complex span	50.93 ± 17.74	60.59 ± 18.63	58.5 ± 20.16	0.003*	0.53	0.815	0.004
ROCFT 30 min	21.77,6.41	25.09,5.76	25.82,4.66	0.001*	0.54	0.717	0.10
RAVLT 30 min	10.23,2.62	10.41,3.5	11.86,1.99	0.707	0.06	0.404	0.18
TMT B (s)	78.95 ± 21.41	75.18 ± 14.82	71.33 ± 35.19	0.335	0.19	0.892	0.03
SPM	6.05 ± 2.01	6.5 ± 2.48	6.29 ± 1.9	0.404	0.19	0.403	0.29
DASS depression	1.5 ± 3.28	1.82 ± 3.02	0.71 ± 1.44	0.405	0.10	0.127	0.36
DASS anxiety	0.5 ± 0.8	0.91 ± 2.04	0.64 ± 1.15	0.323	0.24	0.755	0.11
DASS stress	3.41 ± 3.29	4.27 ± 3.99	13.86 ± 12.01	0.325	0.23	0.069	0.62
*n*-back	17.68 ± 12.56	13.41 ± 10.34	13.86 ± 12.01	0.102	0.37	0.542	0.08
Digit span	14.14 ± 2.59	14.27 ± 3.37	15.07 ± 3.41	0.792	0.04	0.865	0.04
Corsi block	14.45 ± 3.65	14.64 ± 3.47	15.21 ± 3.79	0.786	0.05	0.303	0.19
**MB**
Complex span	58.83 ± 20.52	59.58 ± 16.65	60 ± 18.64	0.796	0.04	0.459	0.18
ROCFT 30 min	21.73 ± 5.76	23.23 ± 6.37	23.47 ± 5.35	0.278	0.25	0.904	0.03
RAVLT 30 min	10.96 ± 3.33	11.54 ± 3.4	11.93 ± 2.52	0.262	0.17	0.224	0.22
TMT B (s)	76.91 ± 33.74	65.08 ± 15.83	61.31 ± 18.48	0.039*	0.38	0.389	0.17
SPM	6.12 ± 1.48	5.88 ± 1.83	6.07 ± 1.39	0.552	0.15	0.922	0.04
DASS depression	1.21 ± 1.61	0.82 ± 1.3	1.27 ± 1.71	0.162	0.26	0.351	0.27
DASS anxiety	1.71 ± 2.44	1.59 ± 2.5	2.13 ± 3.46	0.783	0.05	0.701	0.04
DASS stress	4.42 ± 5.52	4.51 ± 6.02	3.93 ± 5.69	0.889	0.02	0.930	0.01
*n*-back	19.21 ± 11.52	15.38 ± 8.47	14.73 ± 9.82	0.045*	0.36	0.797	0.04
Digit span	16.08 ± 3.19	16.42 ± 3.61	17.13 ± 2.92	0.569	0.10	0.655	0.09
Corsi block	14.83 ± 3.47	14.79 ± 3.45	15.4 ± 2.64	0.943	0.01	0.8	0.06
**Dual *n*-back**
Complex span	53.16 ± 19.93	55.81 ± 18.54	53.25 ± 15.74	0.237	0.14	0.581	0.07
ROCFT 30 min	23.25 ± 5.73	24.17 ± 5.18	26.14 ± 3.53	0.386	0.17	0.074	0.60
RAVLT 30 min	11.54 ± 3.02	12.04 ± 2.51	12.19 ± 2.4	0.306	0.18	0.928	0.02
TMT B (s)	69.49 ± 21.76	80.4 ± 37.41	68.31 ± 16.99	0.099	0.34	0.420	0.23
SPM	6.19 ± 1.98	6.12 ± 2.05	6.62 ± 1.67	0.859	0.04	0.060	0.52
DASS depression	1.58 ± 2.53	1.73 ± 2.81	1.62 ± 2.8	0.733	0.06	0.508	0.14
DASS anxiety	0.65 ± 1.23	0.92 ± 1.16	1.19 ± 2.32	0.215	0.22	0.383	0.17
DASS stress	5.58 ± 6.33	4.77 ± 4.87	5.38 ± 5.23	0.337	0.14	0.868	0.02
n-back	14.81 ± 9.83	7.92 ± 8.86	9 ± 11.05	0.001*	0.73	0.570	0.19
Digit span	15.23 ± 3.46	15.54 ± 3.4	15.56 ± 2.78	0.448	0.09	0.734	0.06
Corsi block	14.38 ± 3.51	15.81 ± 3.26	15.88 ± 3.38	0.070	0.42	0.417	0.29
**Control**
Complex span	56.68 ± 18.69	59.61 ± 16.08	55.47 ± 12.18	0.357	0.17	0.022*	0.32
ROCFT 30 min	23 ± 4.18	23.13 ± 7.59	21.73 ± 6.36	0.902	0.02	0.254	0.20
RAVLT 30 min	11.47 ± 2.2	12.53 ± 2.29	11.93 ± 2.49	0.099	0.47	0.818	0.06
TMT B (s)	77.99 ± 28.22	82.12 ± 44.81	71.48 ± 30.31	0.603	0.10	0.056	0.28
SPM	6.26 ± 2.02	6.37 ± 1.92	6.27 ± 2.05	0.816	0.05	1	0.00
DASS depression	1.47 ± 2.48	2.21 ± 4.04	2.93 ± 6.35	0.163	0.17	0.048*	0.16
DASS anxiety	1.26 ± 2.08	1.16 ± 2.65	1.87 ± 2.97	0.695	0.04	0.017*	0.54
DASS stress	4.11 ± 3.4	4.16 ± 5.21	5.27 ± 5.7	0.946	0.01	0.127	0.16
n-back	12.16 ± 7.89	11.42 ± 8.27	16.13 ± 12.37	0.618	0.09	0.242	0.31
Digit span	14.68 ± 2.93	15.42 ± 3.1	15.6 ± 3.62	0.206	0.24	0.768	0.06
Corsi block	14.47 ± 3.52	14.68 ± 3.97	14.07 ± 3.49	0.805	0.06	0.183	0.17

Notes. *, significance level at *p* < 0.05.

### Analyses of Near and Far Transfer and Additional Outcomes

The linear mixed-effects models applied to investigate the training gains between groups for all transfer tasks showed a significant interaction effect for the TMT form B ($\chi_{\left(1\right)}^{2}$ = 8.06, *p* = 0.04). The contrast, however, revealed no significant difference between any of the groups with the MB^+^ training. All other models yielded no significant interaction effects for near or far transfer measures pre- to post-training. There was a significant effect for session in the RAVLT ($\chi_{\left(1\right)}^{2}$ = 4.89, *p* = 0.03), the ROCFT ($\chi_{\left(1\right)}^{2}$ = 6.54, *p* = 0.01), and the *n*-back task ($\chi_{\left(1\right)}^{2}$ = 19.27, *p* < 0.001) in the linear mixed-effects models testing group differences to the MB^+^ training. Additionally, a significant group effect was present in the *n-back* task ($\chi_{\left(1\right)}^{2}$ = 8.4, *p* = 0.04). For all the other tasks, neither group nor session main effects were present.

Regarding the post-training to follow-up, the linear mixed-effects model showed a significant interaction between group and session for the DASS stress subscale ($\chi_{\left(3\right)}^{2}$ = 19.27, *p* < 0.001). The investigation of the contrasts showed a significant difference at the follow-up session between the control group (*b* = 2.88, *t*_(51)_ = 2.54, *p* = 0.014, *r* = 0.33) as well as the dual *n-back* training (*b* = 2.49, *t*_(51)_ = 2.28, *p* = 0.027, *r* = 0.30) and the MB^+^ training. Furthermore, there was a significant main effect for session in the TMT form B ($\chi_{\left(1\right)}^{2}$ = 4.43, *p* = 0.04) and a significant effect for group in the *n-back* task ($\chi_{\left(1\right)}^{2}$ = 8.98, *p* = 0.03). All other outcome measures showed no effects for the group, session, and interactions between group and session. All linear mixed-effects model outcomes for the interaction effects are reported in Table 5.

**Table 5:** Group × session interaction effects of the linear mixed model for all WM and near and far transfer measures with MB^+^ training as *a priori* contrast.

	Delta		Delta	
	pre-/post-training		post-training/FU	
	χ^2^	*p*	χ^2^	*p*
Complex span composite	7.29	0.06°	2.99	0.39
score
Near transfer
ROCFT 30 min recall	4.38	0.22	5.96	0.11
RAVLT 30 min recall	1.84	0.61	1.89	0.59
TMT B (s)	8.06	0.04*	2.91	0.41
Far transfer
SPM	1.18	0.76	2.59	0.46
DASS depression	4.93	0.18	5.35	0.15
DASS anxiety	1.57	0.66	3.59	0.31
DASS stress	2.09	0.55	8.04	0.04*
Other outcomes of interest
Digit span	0.97	0.81	0.49	0.92
Corsi block	2.46	0.48	1.21	0.75
*n*-back (wrong answers)	4.15	0.25	1.66	0.64

Notes. °, significance level at *p* < 0.1; *, significance level at *p* < 0.05.

The univariate analyses within the MB^+^ group showed a significant improvement on the ROCFT 30-min recall post-training (*t*_(21)_ = −3.8, *p* < 0.05), indicating a transfer effect to this task. All the other within-group comparisons showed no significant changes, neither at pre-training to post-training nor comparing post-training and follow-up. The within-group analyses of the MB training showed a significant decrease in reaction time post-training in the TMT form B (*t*_(23)_ = 2.19, *p* < 0.05) and a significant decrease in wrong answers at the *n*-back test post-training (*t*_(23)_ = 2.12, *p* < 0.05). There were no other training-related changes or changes at the follow-up session. For the dual *n-back* training, a significant decrease in wrong answers in the *n*-back task was found post-training (*t*_(25)_ = 3.83, *p* < 0.001). There were no other changes in the transfer tasks comparing pre- and post-training. There were also no changes between the post-training and follow-up session. Within-group analyses of the control training showed no training-related changes comparing pre- and post-training. However, there was a significant increase in the DASS depression (*t*_(13)_ = 2.19, *p* < 0.05) and the DASS anxiety subscale (*t*_(13)_ = −2.75, *p* < 0.05) at the 3-moth follow-up. There were no other training-related changes or changes between the post-training and follow-up session.

## Discussion

In this parallel group randomized clinical trial, we investigated two computerized WM trainings based on the multicomponent model (Baddeley et al., 1974) with (MB^+^) and without (MB) inclusion of distractor inhibition on their efficacy of improving WM performance and inducing transfer effects. Both trainings were compared with a dual *n*-back training and an active control intervention in healthy old adults. After accounting for practice effects, only the MB^+^ training group shows an improvement in WM capacity tasks. Compared with a model-based, a dual n-back, and a control training, the MB^+^ training shows an overall tendency for superiority in improving WM capacity, which was particularly evident compared with the MB training. The dual n-back, MB, and control groups showed no improvements on WM capacity. Regarding transfer to trained and untrained cognitive functions, the MB^+^ group showed an improvement in visuospatial learning, the MB group an improvement in a processing speed and visuospatial *n-back* task, and the dual *n-back* group an improvement in an untrained visuospatial *n-back* task. In the direct comparison among trainings, transfer effects were only detected to the processing speed and a visuospatial *n-back* task following the MB training. From post-training to the 12-week follow-up, the control training group showed a decrease in performance on WM capacity tasks.

Our results show that although two trainings were developed on the basis of the same theoretical model, only the MB^+^ group, which includes the distractor inhibition component, improved the WM capacity. While the effect was only tendentially significant in the overall comparison between all trainings, this tendency is supported by the univariate analyses, which showed improvement on untrained WM capacity tasks only in the MB^+^ group, even after accounting for practice effects. This finding is in line with previous studies describing improvements on WM tasks following training on filtering efficacy (Shin et al., 2015; Schmicker et al., 2016; Li et al., 2017). In comparison with previous studies that investigated filtering training and WM training separately, our results expand those findings in the sense that distractor inhibition was embedded in the WM training tasks. In addition, we found a near transfer effect to visuospatial learning following the MB^+^ training. Although a previous study on the effects of filtering efficiency training did not find transfer effects to other cognitive functions, improvement specifically on visuospatial WM was described (Li et al., 2017). Since the improvement on visuospatial learning in our study was not present in the comparison with other trainings, further studies are needed in order to investigate far transfer effects of filtering in combination with WM training. Nevertheless, following our results and those of previous studies, the question arises if filtering efficiency could constitute a task-related process of WM training in old age. Indeed, it has been suggested that filtering training seems to benefit WM improvement by increasing selection abilities which could in turn increase the efficiency of memory encoding (Schmicker et al., 2016). Additionally, it has been suggested that older adults use distractors in cognitive tasks as environmental support in order to counterbalance decreasing cognitive performance (Rumpf et al., 2019). In light of the previous suggested association between reduced WM capacity and reduced filtering efficiency in old age (Jost et al., 2011), training distractor inhibition in the context of a WM as implemented by the MB^+^ training could therefore enhance the selection ability during WM tasks and by that facilitate the completion of a complex WM task, which in turn enhances WM capacity.

This mechanism of action could also explain the absence of improvement on WM capacity following the MB training, which did not train distractor inhibition. In the direct comparison of the training approaches, differences between the trainings in regard to the transfer effect were only found for the TMT form B, indicating improved processing speed following the MB training. This effect is also supported by the univariate analyses, where improvement on the TMT form B and, additionally, small improvement on the visuospatial *n*-back task were found. Although the TMT is designed to measure processing speed, it has been described that a simple span task explained the most variance of the TMT form B, indicating a reflection of WM (Sánchez-Cubillo et al., 2009). Additionally, small effects on improvements on simple span tasks following *n-back* training have been reported previously (Soveri et al., 2017). It could therefore be assumed that the MB training with its tasks structured according to simple span affects WM-related tasks; however, only a combination of the MB tasks with distractor inhibition as implemented in the MB^+^ training has the ability to tap WM capacity. Still, in order to understand if the combination of distractor and WM training indeed displays a task-related process, future studies should investigate this combination of filtering and WM training compared with filtering training or WM training alone in order to understand its benefits for WM capacity in old age.

Besides the differences between the two model-based trainings, we investigated the efficacy of the MB^+^ training compared to a dual n-back. Our results showed a tendency for difference between the MB^+^ and the dual *n*-back training in regard to improved WM capacity, which is supported by the univariate analyses indicating an absence of training gains on WM capacity in the dual *n-back* training. Additionally, improvement on an untrained visuospatial *n-back* task was found following the dual *n-back* training. This finding is in line with a meta-analysis which investigated the efficacy of 33 studies and described a moderate effect of transfer to untrained *n-back* tasks and a small effect to other untrained WM tasks, concluding that the transfer effects following *n-back* training remain task specific (Soveri et al., 2017). Likewise, a comparison between a spatial *n-back* and a verbal complex span training showed no training gains to untrained complex span tasks but an improved performance on an untrained *n-back* task following the spatial *n-back* training (Minear et al., 2016). Holmes et al. (2019) correspondingly described an improvement on an untrained *n-back* task but no cross-paradigm transfer to a verbal complex span task following *n-back* training in a direct comparison of both training paradigms. Additionally, a comparison between *n-back* and arithmetic updating training and their effects on updating and complex WM task yielded improvements only in outcome tasks that were structurally similar to the trained function (Linares et al., 2019). Our results therefore support previous studies which concluded that transfer from *n-back* trainings in comparison with other training strategies is task specific and extends those findings by drawing this conclusion in a sample of old adults.

Next to achieving improvements on untrained WM tasks, producing long-term and far transfer effects to other cognitive functions is of main interest. Our results suggest no far transfer from neither model-based (MB and MB^+^) nor dual *n*-back training to reasoning measured by the SPM test. This is in line with previous research, which reported no far transfer to reasoning following WM training (Minear et al., 2016; Sala et al., 2019; Teixeira-Santos et al., 2019) and filtering training (Li et al., 2017). The lack of far transfer effects to reasoning performances following WM trainings may question the importance of WM training in the old population (Sala et al., 2019). However, among other factors that could explain the absent far transfer effects, the type of outcome measure has been suggested as a moderator, since slightly higher transfer effects were described in reasoning abilities measured with the Cattell test vs. the SPM test (Teixeira-Santos et al., 2019). The authors suggest that measures such as the Cattell test with its division in subtests may reflect reasoning more comprehensively and therefore highlight the importance of used measurement to assess training gains. Additionally, a recent study investigated the far transfer effect of WM training in old age by measuring everyday functioning and reported improvements not only at post-test but also at a 6-month follow-up (Borella et al., 2019). These findings indicate that transfer effects can occur and highlight the importance of shifting the focus from investigating improvements on other cognitive tasks toward transfer to daily life activities in future studies.

At last, three methodological aspects in the field of WM training studies should be discussed. First, although practice effects have been described in WM capacity tasks (Scharfen et al., 2018), most WM training studies do not account for them in their study designs. Our results suggest substantial practice effects in complex span tasks as well as a visuospatial *n*-back task after a repeated test administration before training. This is in line with a meta-analysis, which described practice effects in WM tasks and specifically reported larger practice effects in updating, n-back, complex span, and coordination tasks than for simple span tasks, concluding that unfamiliar and challenging cognitive tasks are more subject to practice effects (Scharfen et al., 2018). As one explanation for the occurrence of practice effects, interference of anxiety has been described, which has been found to be reduced the largest after a second administration and reaching a plateau after a fourth administration of cognitive testing (Jendryczko et al., 2019). In our sample, we found reduced scores on all three subscales of the anxiety, stress, and depression scale at the second test administration and no changes in all three scores between pre- and post-training. Therefore, our results support previous findings suggesting that complex span and *n-back* tasks may be perceived as difficult by the participants and may induce stress and anxiety, which could further also be related to the unfamiliarity of the testing situation. Following our results, we suggest that perceived stress and anxiety may be reduced by a second test administration before the WM training and should therefore be taken into consideration in the form of a double-baseline design in WM training studies. This approach could account for practice effects and, hence, help in detecting the true training gains following WM trainings.

Second, the selection of an appropriate control condition displays an issue in WM training studies (Morrison and Chein, 2011; Shawn Green et al., 2019). It has been recommended that active control condition should be used in training studies in order to account for various effects such as familiarity of testing situation and motivation toward training (Morrison and Chein, 2011; Melby-Lervåg et al., 2016); however, these are not properly controlled for expectancy effects (Morrison and Chein, 2011; Boot et al., 2013). In our study, the control condition consisted of a sham intervention with a similar design of the training conditions, performed on the tablet device. A pilot investigation in a separate sample before the clinical trial yielded no differences between the trainings related to expectancy, indicating that the participants had similar expectations toward their improvements. Nevertheless, it has been suggested that mechanistic studies—whose goal is to identify underlying mechanisms—should additionally include a passive control group, which could help in the interpretation of absent differences (Shawn Green et al., 2019). In our study, we did not include a passive control group. Nevertheless, our study design allowed conclusions regarding the mechanism of action, since only distractor inhibition was manipulated in one of the two model-based trainings. Future studies should carefully choose the appropriate control group(s) in order to gain insight in the trained mechanisms.

Third, the variability of assessment tests, training tasks, paradigms, and transfer measures has been suggested to be a severe issue in order to draw conclusions across studies (Pergher et al., 2020). Although comparative studies—such as ours—help to counteract this issue, future studies should find a consensus for the assessment of transfer by using valid and appropriate tasks, which further investigate the application of the training in daily life (Borella et al., 2019; Pergher et al., 2020).

Despite the vast control of methodological issues and theoretical considerations applied in this clinical trial, we have to acknowledge several limitations. First and most importantly, the *a priori* calculated sample size could not be reached due to the early termination of the study because of restrictive measures due to the COVID-19 pandemic. For this reason, the study is underpowered and the conclusions have to be interpreted with caution. This is specifically evident in the interpretation of the long-term transfer measured at the 3-month follow-up. On the univariate level, we could interpret that all training groups remained on their levels except for the control group that showed a decrease on WM capacity at 3-month follow-up, indicating long-term effects on WM capacity improvement in the MB^+^ training. However, only half of the sample reached the follow-up session, and therefore, this effect has to be interpreted with caution. Second, although the univariate analyses showed no improvements on WM capacity, the low power of the study due to the incomplete sample size could additionally account for the lack of significant difference between the active control group and MB^+^ training in the comparison of all trainings. The uncompleted recruitment led to an unbalanced group size and, therefore, limits the conclusions that can be drawn from the described effects.

In conclusion, our study suggests that a model-based WM training in combination with distractor inhibition in the sense of filtering efficacy is a promising approach to induce improvements in WM capacity. Although the study is underpowered, it shows that a rigorous methodological control by accounting for practice effects and choice of adequate control condition can lead to insights on the effects of possible confounding factors. Future studies are needed to investigate the described mechanism of action in large-scale comparative studies and their far transfer effects to activities of daily life. In this way, we can develop efficient training programs and study their transfer effects, whether it is for the enhancement of cognitive functions and their applicability in everyday life or as a basis for effective rehabilitation programs.

## Data Availability Statement

The anonymized raw data supporting the conclusions of this article will be made available by the authors, without undue reservation.

## Ethics Statement

The studies involving human participants were reviewed and approved by Ethikkommission Nordwestschweiz (EKNZ). The patients/participants provided their written informed consent to participate in this study.

## Author Contributions

PZ, DQ, and SM designed the research. EG and PZ conducted the study. PZ performed the statistical analyses and wrote the manuscript. All authors contributed to the article and approved the submitted version.

## Conflict of Interest

SM is employed by company F. Hoffmann-La Roche Ltd. The remaining authors declare that the research was conducted in the absence of any commercial or financial relationships that could be construed as a potential conflict of interest.

## Footnotes

^1^http://developer.android.com/studio/index.html
