# Supplementary material for: Investigation of a Model-Based Working Memory Training With and Without Distractor Inhibition and Its Comparative Efficacy: A Randomized Controlled Trial on Healthy Old Adults
Source: Front Aging Neurosci. 2021 Jun 15;13:682474. doi: 10.3389/fnagi.2021.682474 (PMC8239181; doi:10.3389/fnagi.2021.682474)
Supplement: Supplementary file 1 [file Data_Sheet_1.PDF]

Supplementary Material

1 Supplementary Data

Construction of the MB and MB$^+$ training

Phonological Loop Task. Previous literature suggests that immediate serial recall tests using a small set of digits, letters or unrelated short words are used for the assessment of the phonological loop (67). In these simple span tests, participants hear a short sequence of digits, letters or words which they have to repeat in the correct order. For our phonological training task, thus a simple letter span test was used as the basic paradigm. Since it has been shown that the immediate memory span declines as the item length increases, the number of presented digits is used as a level of difficulty in the MB training. We recorded all letters of the alphabet in German language. Based on the pronunciation of the letters in German, we included the letters A, B, C, D, E, F, G, I, L, M, O, P, R, S, T, U, W, Z in the general letter pool. A random sequence of letters was presented phonologically over headphones and participants were instructed to recall this sequence. After a retention phase of 2.5 s, participants were shown tabs with the written letters in order to enter the heard sequence. There was no time limit for entering the sequence, however, as soon as the correct number of letters was entered, the keyboard disappeared and after a 1 s break a next sequence was phonologically presented. For the MB training, the sequence length started at 1 letter and increased with one additional letter after 3 repetitions of the original sequence length.

However, it has been extensively documented that similar items (E, G, B, D) are less accurately recalled than dissimilar items (A, F, G, U) also known as the "similarity effect" and that irrelevant sounds disturb the recall of the items, known as the "irrelevant noise effect" (35). Although it has been shown that a training on dichotic listening reduced task-irrelevant speech interference (68), no training up to date included task levels based on the irrelevant noise or similarity effect. Thus, the phonological loop training task for the MB$^+$ training consisted next to the increased sequence length of the following two difficulty levels: 1) presence of irrelevant background sounds or not and 2) presentation of dissimilar or similar digits of items. The irrelevant background noises consisted of royalty free "space" sounds (https:/freesound.org) which were selected with the aim of increasing interference to the letter span task. For the similarity level, a second pool of letters was created which included letters that are perceived as similar in German (B, C, D, E, G, P, T, W or K, H, A). An easy

level would therefore start by a small sequence length and dissimilar digits presented without irrelevant sounds. Subsequently, after the presentation of two sequences with dissimilar letters, one sequence was presented with an irrelevant noise during the presentation of the sequence and the retention phase. As a next level, participants heard a sequence of similar letters and additionally the irrelevant noise. The levels of similar letters or irrelevant noises or both are added to the increasing number of letters to induce increasing difficulty of the training.

Visuospatial Sketchpad Tasks. It has been suggested, that the visuospatial sketchpad can further be distinguished in a visual and spatial subcomponent. For this reason, two tasks were implemented to train the visuospatial sketchpad component. Our review showed that visual pattern span test is broadly used to assess the visual subcomponent (36) and was thus seen as the basis for our MB and MB^+ training task. In the pattern span task, a grid with colored and uncolored squares is presented to the participants for a short time span in order to memorize the pattern of colored squares. Participants then have to recall the pattern by filling all squares that were colored in the previous grid in the right positions. This was done accordingly in our training, by presenting a 2x2 (minimum) and with increasing difficulty up to 5x6 square grid (maximum) for 3 seconds. After a short retention phase of 2.5 seconds, an empty grid appeared to fill in the memorized colored squares. Regarding the spatial subcomponent of the visuospatial sketchpad, the most common task used for assessment is the Corsi block tapping test (35,37), which therefore built the basis for our training task of the spatial subcomponent. Visuospatial WM is tested by a tapping-sequence of pegs arranged on a board. Subsequently, participants have to recall the sequence by tapping on the right pegs. In our MB and MB^+ trainings, objects were arranged in a square and flashed in a specific sequence which the participant had to recall in the same order by tapping on the objects. The task improves in difficulty by adding an item to the previous sequence which challenges the memory recall of the sequence.

Already in early experiments it has been shown, that the presentation of an irrelevant picture after the pattern span matrix and during the retention phase leads to a reduction in performance on the pattern recall, suggesting that the passive visual store is accessible by presented interfering visual inputs (69). It has also been shown, that training of filtering efficiency can improve visual WM, however a WM training task combining filtering efficiency and WM does not exist to date (58). Hence, next to the increasing size of the matrix and number of filled squares as done in the MB training, a level of difficulty was created for the MB^+ by adding an irrelevant visually loaded picture during the

retention phase and the recall of the grid squares. Six pictures were selected from the NASA image and video library based on their visual features since it has been described that the content of the picture (densitiy, color palette, unspecifity of content?) influences the irrelevant picture effect. Similar to the pattern span task, it has been shown that an irrelevant task of haptic movement shifts the spatial attention and by that interferes with a spatial WM task such as the Corsi block tapping test (70,71). As an additional level of difficulty for the MB^+ training, we therefore added a haptic irrelevant movement task between the presentation and recall of the Corsi task next to the increasing sequence length of the presented items. Following the presentation of the object sequence, participants were asked to follow lines displayed as star constellations with their finger until the lines disappeared. When the whole figure disappeared, the object arrangement appeared again and they were asked to recall the sequence of the highlighted objects.

Central Executive Task. The central executive has been postulated to be the most important, however least investigated component of the multicomponent model. It has been investigated that the central executive covers four basic capacities: The ability to focus, divide and switch attention as well as the ability to relate content of WM to long-term memory. Due to its importance in the theoretical framework, we developed a task to train the coordinative function of WM. Random word, letter or digit generation tasks or as well dual tasks have been suggested to assess and investigate the central executive (38,39). Random digit generation tasks showed, that by increasing speed of generation the randomness decreases and it therefore has been implied, that the non-randomness seems to include information on the limit of processing capacity. Due to the nature of this kind of task, it seems unlikely to build a training task with levels. Hence, we developed a basic dual task which targets two tasks at the same time, in our case visuospatial sketchpad and the phonological loop, and took this as the basis of the central executive training task. Subjects were presented a specific item which they had to hold in mind and search in a pool of distracting items. With the right hand, they were instructed to tab on the items they had to remember. With increasing difficulty, more distracting items were shown and the speed of presentation increased. At the same time, they were hearing a high or low tone. By placing the left hand on two buttons, they had to indicate which tone they heard while additionally the speed of presentation increased with increasing level.

Episodic Buffer Task. Since it has been shown that the relation of content of WM to long term memory is not only a process which involves the central executive and can rather be explained through binding procedures, the episodic buffer was introduced to the model. The episodic buffer is a separate

storage system of limited capacity using a multimodal code by providing a temporary retention of integrated information (72). The episodic part holds information from other cognitive systems inclusive the WM in so called “scenes or episodes”. It is a buffer, as it displays an intermediary between subsystems with a different code. A review came to the conclusion, that the episodic buffer can be investigated using unimodal or cross modal binding tasks (73). Typically, tasks where letters or words and a spatial location are presented concurrently are used. In our MB and MB^+ training, a spatial order of letters on certain position with or without a shape around them are presented on a stimulus slide. Followed by a blank slide, one letter or word on a random position is presented and participants had to answer, if 1) the word or letter is the same then one on the stimulus slide and 2) if the location is on the same position than on the stimulus slide. The participant has to give an answer only, if both questions can be answered as correct (72,74). This task was used as the basis for the episodic buffer training task. In our training we used letters framed by squares. In order to create levels, the stimulus array was increased by a letter with increasing difficulty.
